# Epidemiological profile of patients with epilepsy attended in an emergency in a psychiatric hospital in 2020

**DOI:** 10.1192/j.eurpsy.2022.1154

**Published:** 2022-09-01

**Authors:** F. Faria, B. Tarifa, B. Maritan, L. Antonio, M. Ricci, G. Filho

**Affiliations:** FAMERP, Department Of Mental Health, São José do Rio Preto, Brazil

**Keywords:** emergency psychiatric, comorbid, Epidemiology, epilepsy

## Abstract

**Introduction:**

According to the Global Burden of Disease study (WHO, 2010), epilepsy is ranked as the second most impacting neurological disorder worldwide, in terms of disability-adjusted life years, and is often associated with psychiatric comorbidities, stigma and high economic costs. This frequent association between epilepsy and mental disorders is a fact, however, they are often underdiagnosed and undertreated in patients with epilepsy, which further reduces the quality of life of this population and induces the demand for psychiatric emergency care.

**Objectives:**

To give the comorbid relevance between epilepsy and mental disorders, this study aims to identify the main psychiatric illnesses associated with patients with epilepsy treated at the emergency of a psychiatric hospital in 2020. In addition to establishing the clinical and epidemiological factors related to this association.

**Methods:**

All patients diagnosed with epilepsy (G40), according to the International Classification of Diseases, who underwent emergency care at the HABM, São José do Rio Preto, São Paulo, in 2020. Epidemiological and clinical data were collected.

**Results:**

There were 7258 consultations, with only 27 as cid G40. 71.4% were male, 55% single and age between 42-49 years old. 47.6% indicated psychiatric comorbidities (cid F06). 23.8% patients with both disorders were attended by psychiatrist.

**Conclusions:**

The concomitant occurrence of psychiatric disorders and epilepsy has significant relevance. However, it is known that the diagnosis of psychiatric disorders in epileptic patients is sometimes late, poorly conducted or even underdiagnosed. Therefore, knowing the profile of patients with epilepsy allows us to identify the factors associated with the concomitant of psychiatric disorders.

**Disclosure:**

No significant relationships.

